# African Swine Fever Virus as a Difficult Opponent in the Fight for a Vaccine—Current Data

**DOI:** 10.3390/v13071212

**Published:** 2021-06-23

**Authors:** Hanna Turlewicz-Podbielska, Anna Kuriga, Rafał Niemyjski, Grzegorz Tarasiuk, Małgorzata Pomorska-Mól

**Affiliations:** 1 Department of Preclinical Sciences and Infection Diseases, Poznan University of Life Sciences, Wolynska 35, 60-637 Poznan, Poland; hanna.turlewicz@up.poznan.pl (H.T.-P.); anna.kuriga@up.poznan.pl (A.K.); 2 AgriPlus, Marcelinska 92, 60-342 Poznan, Poland; Rafal.Niemyjski@agriplus.pl (R.N.); Grzegorz.Tarasiuk@agriplus.pl (G.T.)

**Keywords:** African swine fever, African swine fever virus, vaccine, immunoprophylaxis

## Abstract

Prevention and control of African swine fever virus (ASFV) in Europe, Asia, and Africa seem to be extremely difficult in view of the ease with which it spreads, its high resistance to environmental conditions, and the many obstacles related to the introduction of effective specific immunoprophylaxis. Biological properties of ASFV indicate that the African swine fever (ASF) pandemic will continue to develop and that only the implementation of an effective and safe vaccine will ensure a reduction in the spread of ASFV. At present, vaccines against ASF are not available. The latest approaches to the ASFV vaccine’s design concentrate on the development of either modified live vaccines by targeted gene deletion from different isolates or subunit vaccines. The construction of an effective vaccine is hindered by the complex structure of the virus, the lack of an effective continuous cell line for the isolation and propagation of ASFV, unpredictable and stain-specific phenotypes after the genetic modification of ASFV, a risk of reversion to virulence, and our current inability to differentiate infected animals from vaccinated ones. Moreover, the design of vaccines intended for wild boars and oral administration is desirable. Despite several obstacles, the design of a safe and effective vaccine against ASFV seems to be achievable.

## 1. Introduction

The second decade of the 21st century was marked not only by the COVID-19 pandemic in humans, but also by the African swine fever (ASF) pandemic in pigs (*Sus scrofa domestica*), wild boars (*Sus scrofa*), and other free-ranging suids. African swine fever, caused by African swine fever virus (ASFV), which belongs to the Asfivirus genus and the Asfaviridae family, is a dangerous, fatal viral disease that occurs in domestic pigs and wild boars and has had a significant negative impact on the global swine industry [1,2,3]. While at least 24 ASF genotypes have been identified to be naturally circulating in Africa, all cases of the spread of ASFV since 2007 into Asia and Europe are a part of a single epizootic of genotype II (with the exception of genotype I, which is endemic to Sardinia) [3]. In China, which produces about 53% of the world’s pork, production dropped by 29.1%. Obviously, such losses from the ASF pandemic have tremendous consequences for the international trade of pig products [4].

Currently, there is no registered vaccine against ASF. Disease control is based on the earliest possible diagnosis and culling entire herds with ASFV-infected animals (reviewed in [5]). The laboratory diagnostics of ASF remain under the strict recommendations of the World Organization for Animal Health (OIE) as well as the European Reference Laboratory (EURL) [6]. While early detection and removal of infected animals appear to be essential to eliminating or reducing the risk of virus transmission, preventive culling or depopulation on the farm often lead to a highly emotional situation where animal owners do not see the rationale for such drastic measures [5]. Compliance with the principles of strict biosecurity protocols both at the farm level and through national disease control ordinances is essential to combating the disease and preventing the virus from spreading to uninfected farms (reviewed in [5]). In the face of multidirectional opportunities for virus transmission, pig farms should be isolated from the outside world. However, these strategies are insufficient and difficult or impossible to implement in countries regardless of the available resources [7,8,9]. Controlling ASFV in Africa is critical to diminishing the possibility of the spread of various genotypes of ASFV in the future. Controlling ASFV in Europe, Asia, and Africa seems to be extremely difficult in view of the ease with which it spreads, the high resistance of AFSV to environmental conditions, and the many obstacles related to the introduction of effective specific immunoprophylaxis [7]. However, research around the world is making the development of an effective ASF vaccine more probable.

## 2. Worldwide Occurrence and Spread of ASFV

**Figure 1:**
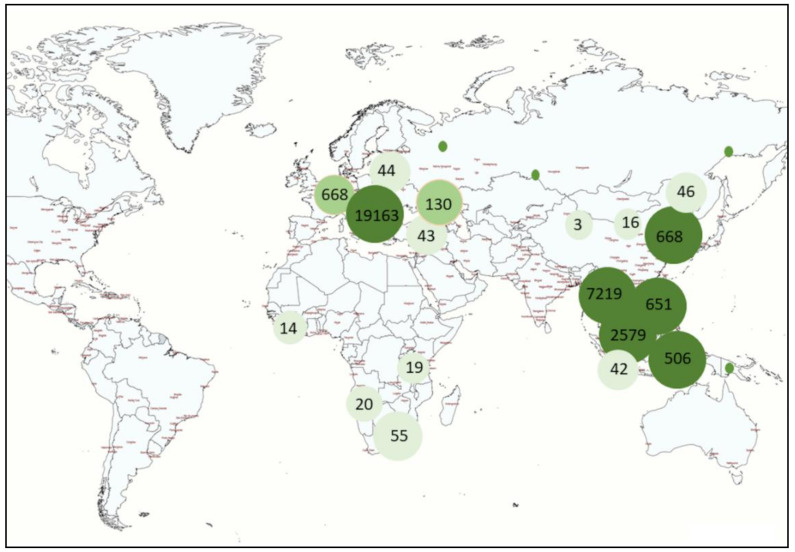
The number of ASF outbreaks around the world from January 2020 to March 2021 [16].

African swine fever was first described by Montgomery in 1921 in Kenya [10]. Since then, the disease has spread. Until June 2007, it had been endemically maintained within the territory of 17 countries south of the Sahara (Angola, Benin, Burkina Faso, Ghana, Guinea Bissau, Cameroon, Kenya, Congo, Madagascar, Mozambique, Nigeria, South Africa, Rwanda, Senegal, Togo, Uganda, and Zambia) and Sardinia [11]. In Europe, ASF was first reported in 1957, when the virus probably spread from Angola to Portugal along with infected feed for pigs [12]. From 1960, the virus rapidly spread to the entire Iberian Peninsula and stayed there for over 30 years [12]. In 2007, “a new era” of ASF emerged when the virus was transferred from Africa to Georgia through the transport of ASF-contaminated slaughterhouse waste on a ship from Africa to the port of Poti [13]. The first cases in Georgia were confirmed in June 2007. Late diagnosis caused the uncontrolled spread of ASF not only within the country, but also to other territories. By the end of 2007, further outbreaks and cases of ASF were recorded in Abkhazia, Armenia, Chechnya, South Ossetia, and the Russian Federation [14,15]. In subsequent years, the virus successfully approached the borders of the European Union (EU) and, in 2012–2014, reached Ukraine, Latvia, and Poland. From January 2020 to March 2021, ASF was recorded in Europe (Latvia, Russia, Hungary, Moldova, Romania, Ukraine, Belgium, Slovakia, Poland, Greece, Germany, and Serbia), Asia (Korea, China, Vietnam, Myanmar, Timor-Leste, the Philippines, Laos, India, and Indonesia), and Africa (South Africa, Cote D’Ivoire, Nigeria, Zambia, and Tanzania) [16]. Table 1 summarizes the cases and outbreaks of ASF that occurred in 2021 [16]. The recent ASF outbreaks are mostly concentrated in Europe, Asia, and Oceania (Figure 1).

## 3. Easy and Multi-Directional Transmission of ASFV

**Figure 2:**
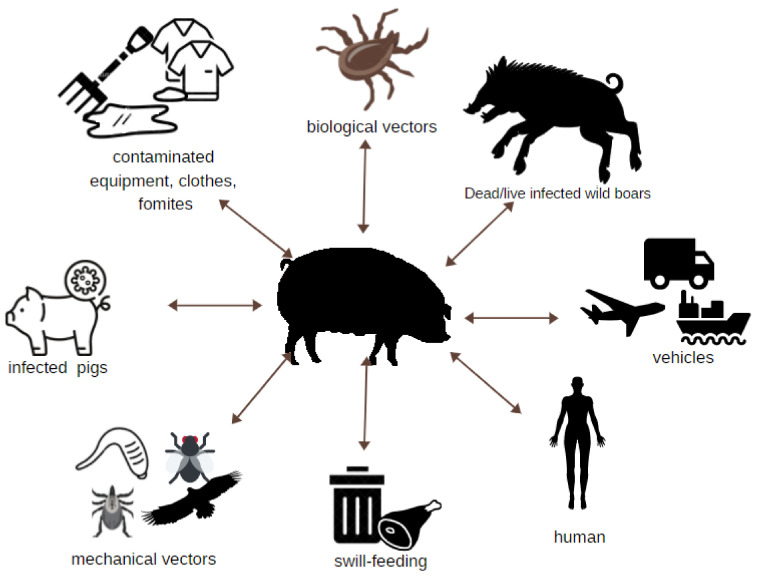
Potential routes of ASFV transmission to pigs.

African swine fever virus can infect domestic pigs directly and indirectly by many routes of transmission (Figure 2), making the provision of farm biosecurity complex and hampering ASF prevention efforts. In ASF epidemiology, two cycles of infection are distinguished: ancient (sylvatic) and new (domestic). Wild boars and wild African suids are involved in the sylvatic cycle [17]. In the domestic cycle, infection occurs only in domestic swine populations, which remain permanently infected and are carriers of the virus. The presence of ASFV has been found in body fluids, feces, and secretions [18].

Asymptomatic carriers are a frequent reservoir of ASFV in South African countries. These include warthogs (*Phacochoerus africanus*) and red river hogs (*Potamochoerus porcus*), also known as river pigs. In this geographical zone, the ASFV vector is a tick species of the genus *Ornithodoros*, family *Argasidae*, known as the soft tick *Ornithodoros moubata* [2,19]. The presence of *Ornithodoros erraticus*, another species of soft ticks involved in maintaining the sylvatic cycle’s transmission, has been confirmed in southern Europe, Spain, and Portugal, where it was an important vector of ASF’s spread during the last century [2]. It is worth emphasizing that ticks infected with the virus can live for up to 5 years and transmit the infectious agent in a trans-stadial (the transition from an immature to an adult tick) or transovarial (from the mother to the offspring) way (reviewed in [20]). The most common European tick (*Ixodes ricinus*) and the meadow tick (*Dermacentor reticulatus*) do not play a role in ASFV transmission and the replication of ASFV in these ticks does not occur [21]. However, these hard ticks may serve as potential mechanical vectors given the fact that the virus can survive in these ticks for six to eight weeks [20].

In Europe, diseased and dead wild boars are the main source of the virus and an extremely important direct or indirect vector of the disease [22]. These animals are highly susceptible to the virulent ASFV genotype II isolates [23]. Wild boars play a major role in the initiation and spread of the disease in Europe, mainly due to their relatively large population and high density, especially in the ASF-affected part of Europe [24]. Research has proven that infection can spread to both swine and wild boars and between these populations [25,26]. Contact between infected wild boars and domestic pigs is a significant factor in the spread of the disease, especially in Eastern Europe (the Caucasus, the Russian Federation), where biosecurity is not sufficient [15]. The ubiquity of wild boar populations in regions with ASF significantly hampers possible efforts to eradicate and control the disease. Moreover, humans contribute to the introduction of the virus into distant areas through various activities. This includes the illegal transport of infected pigs or pork products, which plays a significant role and contributes to the spread of ASFV in Africa, Europe, and Asia [27].

In addition to direct transmission (contact with infected pigs, wild boars, tissue, or a carcass) and spreading through a tick bite, other animals also contribute to ASFV transmission. The unexpected spread of this virus to farms with a high biosecurity level may occur due to the potential transmission of ASFV by flies. Several studies indicate that ASF transmission can occur under experimental conditions after oral intake of fed stable flies (*Stomoxys calcitrans*) [28]. Recent research performed on *Hirudo medicinalis* has shown that leeches may serve as a potential reservoir of ASFV in the absence of common hosts (e.g., pigs and ticks of the genus *Ornithodorus*) [29]. Potential mechanical vectors such as wild or domestic animals that are able to transfer fragments of a carcass or even plant tissues contaminated with the virus should not be neglected. Studies conducted on carcasses of dead wild boar observed three species of predatory mammals and six species of birds to be scavenging on carcasses in different stages of decomposition, including the most common buzzards and ravens. Thus, they confirm the possibility of mechanical transmission of the virus, even over long distances [22].

An important way that ASFV spreads is through the import of contaminated pig products and contaminated items such as feed, equipment, vehicles, and clothing [25]. ASFV can remain viable in animal feed under various environmental conditions, including conditions specific to transatlantic shipping routes [30,31]. Other significant epidemiological factors in ASFV outbreaks are the transportation of contaminated pork products and swill-feeding of domestic swine, which is practiced in the Caucasus, the Russian Federation, and China [15,32]. ASFV has been shown to be highly resistant to temperature, pH, and environmental factors, which are important for transmission to distant areas [33,34,35,36,37,38]. The significantly limited susceptibility of the virus to drying, decay, freezing, and thawing makes it stable in the environment for a very long time [39]. The virus remains contagious in frozen meat for over 1000 days, chilled meat for over 500 days, a rotting corpse at room temperature for up to 18 months, bone marrow for over 6 months, and spleen buried in the ground for 280 days [38]. It has been reported that ASFV can survive in white and Iberian hams for 140 days, in Iberian loins for 112 days, and in Parma ham for 399 days [35,36]. ASFV was also detected after 18, 60, and 83 days of curing in Italian salami, pork belly, and loin, respectively [37]. Thus, meat products can remain an important reservoir of ASFV. The virus, along with such products, often transported over long distances, may appear in new areas where it has not been detected before.

Taking into consideration the biological properties of ASFV and its high resistance to physical and chemical factors, it is highly likely that the ASF pandemic will continue to develop and only the implementation of an effective and safe vaccine will ensure a reduction in the spread of ASFV. Countries located on the European continent and in Asia, neighboring those in which ASF occurs, seem to be the most threatened. However, the disease is also likely to occur in other countries and continents that intensively trade for pigs and pork products, e.g., Canada and the United States.

## 4. Vaccines against ASF—A Short Review and the Latest Achievements

At present, there are no safe and effective vaccines against ASF. Most vaccines that are currently available in veterinary or human medicine are based on the use of inactivated (killed) and live attenuated (weakened) technologies. Inactivated ASFV vaccines fail to provide a protective immune response, which was recently re-confirmed by Blome et al. [40]. While antibodies were developed against the virus, inactivated virus preparations provided no significant protective effect. These preparations were not effective at inducing the specific cytotoxic CD8+ T-cells crucial to the elimination of virus-infected cells [41]. Animals that survived acute infection with a moderately virulent ASFV strain exhibited long-term protection against infection with homologous ASFV strains [42,43]. In addition, administration of colostrum or antibodies from ASF convalescent pigs provided partial protection against experimental challenge with lethal strains of ASFV [44,45]. These facts indicate that designing an effective vaccine appears to be feasible [46]. However, antibodies induced by both viruses and vaccines are not fully able to neutralize ASFV [46]. The location and function of all components that participate in the immune response against ASFV are also not fully understood. Interestingly, some studies have shown that ASFV-specific antibodies, in addition to their protective role, may also cause deterioration during the course of the disease and the occurrence of chronic lesions. The simultaneous presence of the virus and antibodies was correlated with the deposition of immune complexes (e.g., necrotic foci in joint swelling) [47,48,49]. An antibody-related exacerbation was also observed after immunization with attenuated or subunit vaccines [50,51,52]. This phenomenon requires further research [7]. Several previous studies indicate that ASF vaccines should be able to induce both specific antibody and CD8+ T-cell responses to confer appropriate protection [44,53]. Moreover, ASFV is able to avoid the host’s immune response, negatively affecting its immune system and leading to effective virus replication in various ways (reviewed in [46]). The virus is simultaneously capable of establishing complex immune regulation and effective immune system evasion, so ASFV carries a variety of factors that affect the host’s immune system and allow it to establish efficient replication [46,54]. The latest approaches to the ASFV vaccine’s design are focused on the development of modified live vaccines by targeted gene deletion from different isolates or subunit vaccines, with varying results [55].

### 4.1. Live Attenuated Vaccines

Live attenuated vaccines (LAVs) can be based on naturally occurring ASFV strains with reduced virulence or virulent strains attenuated by the engineered deletion of virulence factors [55]. Experimental LAVs based on strains isolated from ticks or naturally attenuated isolates from chronically infected pigs were found to provide homologous protection; however, the final outcome and level of protection depended on several variables, and an ideal vaccine has yet to be constructed [56]. The ability to engineer ASF through homologous recombination or, more recently, CRISPR/Cas9 gene editing enables the construction of specific gene mutants as candidate LAVs that can be tested and ultimately driven toward commercialization. Newly developed, promising vaccines are summarized in Table 2.

Although LAVs have been reported to be promising, the possibility of transmission of the vaccine ASFV is worth mentioning and might have both negative and positive effects in the ASF-affected population. In Barasona et al. [61], the majority of contact sentinel animals developed an immune response, which implies that LAVs induce protective responses not only in directly vaccinated animals, but also possibly in susceptible contact animals through horizontal transmission of the vaccine virus.

The rational deletion of virulence genes and interferon inhibitors appears to be a promising path to obtain candidates for an effective vaccine against ASFV. Examples of virulence-related genes that have been deleted in various studies include 9GL (B119L), UK (DP96R), CD2v (EP402R), DP148R, and different members of multigene families (MGFs) [64]. Nevertheless, it is known that the genetic background of the virus influences the phenotype of the deletion mutant [65]. Deletion of the 9GL (B119L) gene in the highly virulent ASFV isolates Malawi Lil-20/1 (Mal) and Pretoriuskop/96/4 (Δ9GL viruses) resulted in complete protection against parental isolates [62]. Unfortunately, when a similar deletion was used to attenuate ASFV Georgia 2007, attenuation was achieved, but the protective and lethal doses were similar. Full attenuation was achieved through additional deletion of DP96R (UK) [62,63,66].

Despite the above-described data, rational deletion does not always produce the intended effects. For example, infection with a naturally attenuated OURT88/3 strain induced protection against the same or closely related strains but simultaneously caused side effects. Reis et al. [60] attempted to increase the vaccine’s safety by deleting the I329L gene. Surprisingly, the protection induced by the OURT88/3ΔI329L deletion mutant was significantly reduced against challenge with the virulent OURT88/1 isolate. A decrease in the level of antibodies against ASFV protein VP72/B646L and the number of IFN-γ-producing cells in the blood was observed in unprotected animals, while the concentration of IL-10 in the serum of animals immunized with OURT88/3ΔI329L was increased. The same study also showed that the deletion of the I329L gene fails to attenuate the virulent Georgia/2007 isolate [60]. Previous studies showed that the deletion of another IFN inhibitor (A276R) from the NH/P68 isolate resulted in a complete loss of protection against Arm07 challenge, as opposed to the complete protection provided by vaccination with wild-type NH/P68 [67]. The next example of the unpredictability of genetic manipulation involving simultaneous deletion of multiple genes from the ASFV genome is the study conducted by Gladue et al. [68]. The authors showed that the deletion of CD2-like and C-type lectin-like genes significantly decreased the protective potential of the experimental vaccine strain attenuated following the deletion of 9GL from the Georgia 2010 isolate (ASFV-G-∆9GL). The deletion of the EP402R ORF, encoding CD2-like protein (CD2v), and/or the EP153R ORF, encoding viral C-type lectin-like protein, in the 9GL-deleted background resulted in ASFV-G-Δ9GL/ΔCD2v and ASFV-G-Δ9GL/ΔCD2v/ΔEP153R, which harbor two and three gene deletions, respectively. After these gene manipulations, viremia levels induced by ASFV-G-Δ9GL/ΔCD2v and ASFV-G-Δ9GL/ΔCD2v/ΔEP153R were almost undetectable when inoculated in domestic pigs and did not protect animals against challenge with the parental virulent strain ASFV-Georgia, in contrast to ASFV-G-Δ9GL, which provided robust protection during challenge. ASFV-G-Δ9GL/ΔCD2v/ΔEP153R also had a decreased ability to replicate in vitro in swine macrophage cultures when compared with parental ASFV-G-Δ9GL [68].

These studies confirm that the results of ASFV genetic modifications may be unpredictable and experimental validation of new strains is essential in any case. Moreover, the results suggest that the rational development of new ASFV vaccines requires great caution and avoidance of the direct transfer of results obtained by specific gene deletions in a given virus strain to a new field of isolates [68].

A significant discovery in recent years was the gene-deleted, attenuated strain HLj/18-7GD constructed by Chen et al. [59]. According to the study, the HLJ/18-7GD strain, with the deletion of seven genes, was unable to revert to a virulent phenotype and provided complete immunity against a lethal ASFV challenge in pigs [59]. These results are very promising for ASF vaccine development. At this point, however, it should be noted that the authors did not present the whole NGS sequence of virus-DNA-lacking genes. The study also did not check for the absence of the wild-type virus in tissues of vaccinated animals at longer times after challenge (more than 1 month), which is an important safety concern.

In 2015, O’Donell et al. [69] derived the recombinant virus ASFV-G-ΔMGF from the highly virulent ASFV Georgia 2007 isolate. This was the first experimental vaccine reported to induce protection in pigs challenged with the highly virulent ASFV Georgia 2007 isolate and provided further support to the role of MGF genes in ASFV virulence. The researchers deleted six genes belonging to MGF360 or MGF505: MGF505-1R, MGF360-12L, MGF360-13L, MGF360-14L, MGF505-2R, and MGF505-3R. ASFV-G-ΔMGF turned out to be completely attenuated in swine. Pigs inoculated intramuscularly (i.m.) with either 10^2^ or 10^4^ 50% HAD_50_ did not exhibit signs of the disease. After challenge with the highly virulent parental ASFV strain, animals did not develop clinical signs, although a proportion of these animals harbored the challenge virus.

In a study by Chen et al. [59], China’s first ASFV isolate, Pig/Heilongjiang/2018 (HLJ/18), was chosen as the foundation for developing a live attenuated vaccine. In this study, six new mutants were created by removing segments of genes encoding one to seven distinct proteins that have previously been shown to be important for the virulence of various ASFVs using a homologous recombinant DNA technique. The most important of the gene-deleted viruses was determined to be HLJ/18-7GD (in which the genes encoding MGF505-1R, MGF505-2R, MGF505-3R, MGF360-12L, MGF360-13L, MGF360-14L, and CD2v have been deleted). To evaluate the efficacy of the gene-deletion virus in inducing protective immunity, pigs were challenged i.m. and orally with a virulent ASFV strain. The pigs vaccinated twice with 10^5^ TCID_50_ with HLJ/18-7GD were exposed to the lethal HLJ/18 at a dose of 200 PLD_50_ i.m. two weeks after the second vaccination or p.o. at a dose of 10^6.5^ HAD_50_ 3 weeks after the second vaccination. All vaccinated pigs survived the 3-week observation period. Low levels of viral DNA were detected in the lymph node in one pig in the i.m. challenged group. Viral DNA was not detected in any of the pigs in the orally exposed group. The duration of protection following administration of this vaccine was also assessed: six pigs were vaccinated with one dose of the 10^6^ TCID_50_ vaccine and then exposed to the lethal virus 10 weeks after vaccination. It was found that all vaccinated pigs remained clinically healthy and survived the observation period and most pigs had no viral DNA. It is possible that the long-term immunity induced by a single dose of 10^6^ TCID_50_ from HLJ/18-7GD can provide solid protection against exposure to lethal ASFV, and that two administrations of 10^6^ TCID_50_ from HLJ/18-7GD may adequately protect pigs throughout life [59].

The safety of administration of HLJ/18-7GD in pregnant sows was also assessed in the above study [59]. Sows were vaccinated with 10^6^ TCID_50_ HLJ/18-7GD on days 35, 63, and 94 of gestation and monitored for up to 4 weeks postpartum. All sows remained clinically healthy and gave birth to their piglets on the expected dates, indicating that vaccination with HLJ/18-7GD is unlikely to cause complications or miscarriage in pregnant sows or affect piglets’ health. These results suggest that the HLJ/-18-7GD ASFV vaccine is both safe and effective, and might play a key role in limiting the virus’s spread in the future [59].

Despite the very promising results of the above study, HLJ/18-7GD may prove to be ineffective in Europe, where the most serious problem is the reservoir in wild boars. On the contrary, in Asia ASFV is mainly a problem in domestic pigs. Extensive research is required before HLJ/18-7GD can be placed on the European market. This strain would have to provide protection against the virus variants circulating in Europe, and research is also needed to confirm the lack of a carrier state following vaccine administration. Importantly, this vaccine requires evaluation of the effectiveness and safety after per os administration in wild boars, which is a key factor in the disease’s eradication.

Another promising novel LAV candidate was proposed by Borca et al. [57]. The authors discovered that removing the I177L gene from the ASFV resulted in sterile immunity against the Eurasia strain. ASFV isolates from domestic pigs, wild boars, and ticks from Africa, Europe, and the Caribbean were sequenced to assess ASFV I177L protein conservation. The I177L is a gene, transcribed during the late phase of viral infection, that encodes a protein of unknown function. According to the study, removing the previously unknown I177L gene from the highly pathogenic ASFV Georgia 2010 strain (ASFV-G) resulted in a virus (ASFV-G ΔI177L) that was fully attenuated in pigs [57]. In this study, pigs vaccinated with single doses (10^2^–10^6^ HAD_50_) of the ASFV-G-ΔI177 L strain remained clinically healthy throughout the observation period of 28 days, regardless of the vaccine dose. A slight viral load was observed in vaccinated animals at 28 dpi, but they did not show clinical signs of ASF. Animals inoculated i.m. with ASFV ΔI177L produced a robust virus-specific antibody response and were fully protected when faced with virulent parental ASFV-G over a 28-day observation period. Additionally, shedding to sentinel animals during the 28 days of cohabitation was not observed, indicating that transmission of ASFV-G-ΔI177L from infected to naive animals is barely possible [57].

ASFV-G-I177L is one of the few modern vaccine candidate strains that has been shown to induce protection against the ASFV Georgia isolate, and is the first vaccine capable of inducing sterile immunity against the ASFV strain involved in the recent outbreaks [57]. Since the deletion of any other gene related to suppression in other ASFV isolates failed to effectively suppress ASFV-G, the discovery of suppression by deletion of the I177L gene is surprising, even though the host pathways that mediate defense against ASFV infection are still being debated [57].

### 4.2. Vectored Vaccines and Subunit Vaccines

The essence of vectored vaccines is the inclusion of genes from a pathogenic microorganism in the vector genome [70]. Genes originating from the pathogenic microorganisms encode protective antigens against a targeted disease. A vaccinia virus was used early on as a viral vector, and as a result of vaccination both humoral and cellular immunity were described to have developed [71]. Safety in this type of vaccine is ensured by removing or replacing virulence genes or by preventing virus vector replication [70]. Importantly for the future development of a vector-based vaccine against ASFV, viral vectors enable the encoding of immunogens that can serve as vaccine markers, making it possible to differentiate infected animals from vaccinated ones (DIVA) [70]. To date, Pseudorabies virus (PRV), vaccinia virus Ankara, alphaviruses, and adenoviruses have been used as vectors for recombinant live ASFV vector vaccines in experimental studies [72,73,74,75,76].

In 2013, BacMam-sHAPQ, the baculovirus-based vector, was used for the delivery of the sHA/p54/p30 fusion construct to pigs and provided protection against a sub-lethal challenge in four out of six pigs [77]. Three years later, a recombinant Newcastle disease virus (rNDV) expressing ASFV protein 72 (p72) was evaluated [78]. In this study, mice were immunized with rNDV/p72. Animals developed high titers of ASFV-p72-specific IgG antibodies and had higher levels of IgG1 than IgG2a. T-cell proliferation and the secretion of IFN-γ and IL-4 were also elicited [78]. Recently, Feng et al. [72] constructed a PRV-attenuated strain expressing ASFV CD2v protein (PRV-ΔgE/ΔgI/ΔTK-(CD2v)) by the CRISPR/Cas9 technology and inducing CD2v-specific humoral and cellular immune responses in mice. Further studies are needed to confirm the effectiveness of these modified strains in pigs and to evaluate their potential for preventing ASF [72]. Alphavirus replicon particles (RPs) expressing p30, p54, and p72 have also been used to immunize pigs [79], albeit the study did not include data on cell-mediated immune responses and protection against virulent challenge [70]. Nevertheless, vaccinated pigs developed a strong antibody response against p30 and low levels of anti-p54 antibodies. No antigen-specific antibodies were detected in sera of p72-immunized pigs, although the addition of the sHA domain of CD2v in p72 resulted in detectable levels of antibodies against p72.

Antigen cocktails delivered by virus vectors are another type of vectored vaccine being tested in pigs as a vaccine against ASFV. One of these studies used an adenovirus, which delivered ASFV antigens p30 + p54 + p72 + pp62 and ASFV genes A151R + B119L + B602L + EP402R∆PRR + B438L + K205R + A104R. The other study was based on modifications of the Vaccinia virus Ankara-vectored ASFV antigens p72, CD2v, and C-type lectin; however, none of these immunization studies was tested against virulent virus challenge [74,75,76].

Subunit vaccines contain only purified, immunogenic antigens that are part of the cells of pathogenic microorganisms and specifically stimulate the immune system to produce specific antibodies [80]. These vaccines are most often based on proteins, peptides, or polysaccharides that contain immunogenic epitopes that elicit an immune response [80]. Finding an effective antigen for a subunit vaccine is not straightforward [80]. As previously thought, ASFV is unable to induce neutralizing antibodies, whereas many decades of research on convalescent pig serum have allowed for the discovery and description of several ASFV proteins (p30, p54, p72, CD2v, EP153R, p12, D117L, and pp62) [4]. P72, P30, and P54 are considered to be the most significant antigenic proteins that cause humoral immune responses during infection. Virus adsorption can be prevented by antibodies to P72 and P54, and virus endocytosis can be preserved by antibodies to P30 [17]. These discoveries have led to a further focus on subunit vaccine research. Nevertheless, antibody-mediated neutralization in relation to ASFV infection remains controversial [7]. Unfortunately, in some studies, the protection provided by these proteins could not be restored in other infectious models [81] and their use in experiments with vaccines for virulent strains of ASFV did not yield significant effects [51,81,82,83]. One method for assessing ASFV protective antigens and their neutralization sites has been to express them in cells, then present them to antigen-presenting cells to induce strong anti-ASFV neutralizing antibodies [17]. Due to its high level of expression, low cost, and high degree of protection in disease therapy, the subunit vaccine has been extensively studied in recent decades [50,51,52,73,80,84].

Recently, Goatley et al. [73] performed an interesting and promising study on a subunit vector-based vaccine against ASF. They identified the induction of ASFV-specific antibody and cellular immune responses by different viral-vectored pools of antigens selected based on their immunogenicity in pigs. In this study, a pool of ASFV genes was used and vectored by replication-deficient human adenovirus 5 (rAd) prime and a modified vaccinia Ankara (MVA) boost encoding the same antigen. Following vaccination with one of these pools, which included eight virally vectored ASFV genes (B602L, B646L/p72, CP204L/p30, E183L/p54, E199L, EP153R, F317L, and MGF505-5R), 100 percent of pigs were free of fatal disease when challenged with a usually lethal dose of virulent ASFV [73]. In these studies, two experiments were conducted in succession, with positive findings in the second experiment, where six out of six pigs were protected from lethal doses of the virulent genotype I of ASFV strain OUR T 88/1. The number of survivors after vaccination with the same antigen pool differed between the two experiments, possibly because the immune dosage of the recombinant viral vector was raised and an immune system overreaction was treated with flunixin meglumine (a non-steroidal anti-inflammatory drug) on day 5 after challenge, relative to experiment 1, which protected two out of six pigs from lethal doses of the virulent OUR T 88/1 isolate [4]. Although all vaccinated animals were viremic and developed disease symptoms, the proposed combination offers hope for the development of an efficacious subunit vaccine.

In 2020, Urbano and Ferreira proposed a new protein, identified in the ASFV nucleoid, that may have the potential to be used in subunit vaccine design. pA104R is one of the proteins previously demonstrated to be the main targets of serological immunity in pigs [85]. This protein appears to be profoundly involved in the spatial organization and packaging of the ASFV genome. The antibody response to pA104R is higher in asymptomatic than in chronically infected animals; hence, antibodies against this protein might be an indicator of an effective immune response and are probably involved in protection. Urbano and Ferreira [86] suggest that pA104R may be included in subunit vaccine formulations and has some potential to confer protective efficacy. However, deletion of the pA104R gene from the non-pathogenic Ba71V by Freitas et al. [85] reduced the replication of Ba71VΔpA104R such that it could no longer be isolated. The authors speculate that very low expression of pA104R made the replication of the mutant virus impossible, and that the mutant virus probably required a more stable cell line.

In Pérez-Núñez et al. [84], immunization of pigs with a combination of several viral DNAs and proteins resulted in activation of the cell-mediated immune response. Selected combinations of ASFV recombinant proteins (p15, p35, p54, CD2v-E, and p72) and pcDNA-expressing ASFV genes were chosen to assess their ability to induce humoral and cellular immune responses in pigs. Induction of antibodies and a specific cell-mediated immune response was measured with IFN-γ ELISpots. The ability of serum antibodies to neutralize infection in vitro in some vaccinated animals imparted dignity to specific viral antigens used in the immunization. These data may help with the development of future subunit vaccines against ASFV [84].

## 5. Concerns about Vaccines

### 5.1. African Swine Fever in Wild Boars

African swine fever virus is only able to infect domestic pigs, wild pigs, and ticks, which may be a limitation in the work on obtaining a harmless and effective vaccine against ASF due to the lack of an animal model other than the natural host. The introduction of an effective vaccine into general use is especially important with wild boars. Wild boars are highly susceptible to ASF, which is undoubtedly an additional obstacle in the prevention and control of the disease in Europe [87,88]. Wild boars are a major source of the spread of ASF in the current epizootic in Europe. In Africa, warthogs play a crucial role in transmitting ASFV. In all European countries affected by ASF so far, this disease was probably introduced to the country and later to the domestic pig population directly or indirectly through wild boars [88]. Currently, hunting and population control are the primary means of mitigating the spread of ASF from its primary reservoir. However, according to the European Food Safety Authority (EFSA), controlling the wild boar population only by hunting or sanitary culling does not always yield the expected results (an appropriate reduction in the wild boar population). In most European countries, the population of this species of animals grew significantly in many regions until the appearance of ASF despite hunting [89]. Thus, other management strategies to stop further population increases have been suggested, such as strictly avoiding supplementary feeding. Under good environmental conditions, reducing juvenile survival will have the largest effect on the population growth rate, whereas strong hunting pressure on adult females will lead to the most effective population control in years with poor conditions [89].

Vaccines intended for wild boars should be tested strictly on this species, and it is also desirable to administer them in the form of bites available to boars in the natural environment. Since vaccination of wild boars by injection is not a viable option, an oral vaccine would be recommended due to its proven efficacy in the past in eradicating classical swine fever in wild boars [54]. Unfortunately, most of the studies published so far utilized injected vaccines. Taking all of this into consideration, the chances of limiting the spread of ASFV among wild boars by vaccination are supremely limited. In the next few years, this scenario is unlikely to occur [90]. Few studies have been carried out on the oral immunization of wild boars against ASF [58,61]. The first report on the experimental vaccination of wild boars against genotype II ASFV, and also the first study on oral immunization against any ASFV strain in wild boars, was published in 2019 by Barasona et al. [61]. The use of a naturally attenuated ASFV isolate (Lv17/WB/Rie1217) provided 92% protection after inoculation with the virulent ASF virus isolate Arm07. None of the vaccinated animals developed clinical signs or serious side effects following challenge. However, only a small group of animals was used for the study (12 vaccinated animals). The study showed that orally vaccinated animals can shed the vaccine virus, which, on the one hand, can help to increase the vaccination coverage, reducing the need for large-scale production and administration of vaccines in the field. On the other hand, shedding the vaccine virus may mean that wild boars may be persistent carriers of the virus [61].

As previously mentioned, ASFV-G-∆I177, obtained by deletion of the I177L gene from the genome of ASFV-G, has been proven to be safe and highly efficacious in challenge studies using parental ASFV-G [57]. Borca et al. in 2021 performed a new study administering this strain by the oronasal (o.n.) route and achieved a similar efficacy to that of i.m. administration. o.n. inoculation of a high dose of ASFV-G-∆I177L did not result in the appearance of clinical signs of ASFV until the day of the challenge at 28 days post-infection (dpi). Viremias induced in o.n. inoculated animals were lower throughout the experimental period than those measured in animals vaccinated i.m. Despite concerns that the low viremia titers measured after o.n. administration could weaken the protection, animals inoculated o.n. with ASFV-G-∆I177L remained clinically healthy after challenge with 10^2^ HAD_50_ ASF-G during the 21-day observational period. Moreover, the ASFV-specific antibody responses, mediated by IgG1 or IgG2, observed in animals inoculated by the o.n. or the i.m. route did not differ. Therefore, the ASFV-G-∆I177L vaccine candidate was found to be effective after both o.n. and i.m. administration after challenge with the virulent parental ASFV-G. What is important, researchers suggest, is that o.n. inoculated animals did not shed enough virus to infect naive pigs during 28 days of cohabitation. These facts indicate that o.n. administration of ASFV-G-∆I177L is safe and facilitates the potential use of this vaccine in wild swine populations [58].

### 5.2. Complex Nature of the Virus

The inability to construct an effective vaccine is also due to the complex structure of the ASFV. It encodes a wide variety of proteins related to host immune evasion and exhibits a wide range of modulation mechanisms aimed at the host’s innate immune system [62].

ASFV is able to replicate in macrophages despite the extremely unfavorable environment inside these cells [91]. By avoiding detection and inhibiting host defense mechanisms, ASFV replicates rapidly and causes clinical disease in pigs. Inhibition of the induction of IFN type I in response to infection with virulent ASFV is essential to rapid viral replication [60,62]. It is suggested that the deletion of genes that inhibit the induction of IFN type I may debilitate the virus [60]. ASFV, similarly to numerous other viruses, encodes anti-apoptotic proteins that prevent the destruction of infected cells. Among these proteins, A179L, A224L, EP153R, and DP71L have been distinguished [92,93,94,95,96]. Discovering all or most of them will enable the rational deletion that is associated with vaccine design. It also remains important to thoroughly understand the purposes and functions of multigene families (MGFs) in modulating host immunity [60]. MGFs are considered to be IFN modulators and therefore may serve as potential targets for the development of rationally attenuated vaccines [97,98]. Furthermore, the identification of mechanisms related to host immune impairment and an understanding of the immune protection determinants are critical to the development of an effective vaccine [62]. The complexity of these processes is indicated, for instance, by the fact that ASFV encodes more than 80 structural proteins [99], which play crucial roles in virus attachment, entry, and replication [100]. In addition, the broad-spectrum virulence of many ASFV strains significantly hinders the identification of immune protection determinants [62]. Another important issue in ASFV–cell–host interactions is the ASFV-induced control of important mechanisms, such as the activation of cellular transcription factors, pro-inflammatory factors, and cytokines through the expression of ASFV-encoded genes (e.g., A238L). These phenomena enable ASFV to evade the host immune response [101,102,103,104]. Another major difficulty is the variability in the vaccine and field ASFV strains. ASFV is diverse with respect to genotypes—24 have been identified to date and the vaccine should optimally cover all of the genotypes, which is an important aspect in the event of a potential escape of another genotype from Africa; hence, it would not be necessary to develop the vaccine anew [7,105,106,107].

LAVs provide protection against a homologous ASFV challenge in most cases, although cross-protection, as a multifactorial phenomenon that depends on numerous variables, remains a challenge. Little attention has been paid to the 24 genotypes endemic to Africa, as most of the research concerns the genotypes circulating in Europe and Asia [18,105,106,107]. Vaccines that target ASFV genotypes in Europe or Asia are unlikely to show cross-protection against African endemic strains. Lopez et al. [108], analyzed the cross-protection between phylogenetically distant strains using BA71∆CD2, a recombinant live attenuated virus lacking CD2v (the ASFV hemagglutinin). They showed that five out of six pigs immunized once with BA71∆CD2 survived the tick-bite challenge with the RSA/11/2017 strain (genotype XIX, clade D) and only two out of six pigs in the study survived the challenge with the Ken06.Bus strain (genotype IX, clade A), which is phylogenetically more distant from BA71∆CD2 than the RSA/11/2017 strain. The researchers also observed that homologous prime-boosting with BA71∆CD2 improved the survival rate to 50% after Ken06.Bus challenge, while 100% of the pigs immunized with BA71∆CD2 and boosted with the parental BA71 virulent strain survived a lethal challenge with Ken06.Bus, barely exhibiting the clinical signs of the disease [108]. The cross-protection may depend not only on the sequence similarity, but also on other still-unknown factors, which certainly require further research.

### 5.3. Lack of an Established Macrophage Cell Line

An established, licensed porcine macrophage cell line is necessary for commercial production of future ASFV LAVs [109]. The development of most vaccine candidates is hindered by the lack of a continuous cell line that is adequately susceptible to ASFV while avoiding further genetic adaptations within the ASFV genome [46]. Obtaining primary macrophages is laborious and expensive, and contamination may lead to cell wastage [110,111].

The above-mentioned obstacles have partially been overcome by the adaptation of several virulent field isolates to grow in Vero or MS cells, although the cell culture adaptation’s growth resulted in viral genome rearrangements [56,112,113]. It is difficult to assess the effects of the variable point mutations observed during the adaptation of ASFV to Vero cells, which may result in phenotypic changes in the virus [114]. Chen et al. [59] found that primary bone marrow cells (PBMs) were suitable for the growth of HLJ/18-7GD. The researchers indicate that at least 200,000 doses of the vaccine (10^6^ TCID50/dose) can be produced from PBMs derived from one specific pathogen-free piglet. Although they do not form a cell line, PBMs are likely to enable mass production of the vaccine [59]. Furthermore, large-scale vaccine production using primary cells from animals is not ethically acceptable or feasible. Sanchez et al. [115] characterized four porcine cell lines (IPAM WT, IPAM-CD163, WSL, and CΔ2+) with regard to their macrophage surface marker phenotype, susceptibility to ASFV infection, and virus production capacity. Two different ASFV isolates, Armenia/07 and E70, and the attenuated NHV/P68 were used. The results of the study revealed that cells used in the study expressed only low levels of specific receptors linked to the monocyte/macrophage lineage, with low levels of infection overall, with the exception of WSL, which showed more efficient production of strain NHV/P68 but not of strains E70 and Armenia/07 [115].

ASFV-G-ΔI177L proved to be safe and highly efficacious in challenge studies using parental ASFV-G in the previously described studies by Borca et al. [57,58]. Effective replication of this vaccine strain was possible only in primary swine macrophages, which limits the large-scale production potential. Recently, the same research team proposed an ASFV-G-ΔI177L derivative strain with a spontaneous deletion after cell passages in the left variable region (ASFV-G-ΔI177L/ΔLVR) that efficiently replicates in a stable porcine cell line [116]. This deletion allows for growth in stable cultures while maintaining the potency and efficacy of the parental vaccine strain. ASFV-G-ΔI177L/ΔLVR maintained the same level of attenuation, immunogenic characteristics, and protective efficacy as the previously presented ASFV-G-ΔI177L in challenge studies. This discovery allows us to overcome one of the major obstacles to the manufacture of an ASF virus vaccine, which depends on the production of vaccine in primary swine macrophages. ASFV-G-ΔI177L/ΔLVR is the first ASF vaccine candidate that allows us to retain the vaccine characteristics while simultaneously replicating the vaccine in lab-grown cell cultures and large-scale commercial vaccine manufacturing, features that are extremely important to vaccine production and ultimately disease eradication [116].

### 5.4. Safety

Another important aspect is the safety of the vaccine used in pigs. A major problem with LAVs is the potential for the vaccine virus to revert to a virulent strain when replicating in vaccinated animals. Previously developed live ASFV vaccines, attenuated by serial passages in a cell culture or by using naturally isolated avirulent strains, turned virulent and caused 10–50% mortality when used in breeding pigs in Portugal and Spain in the 1960s [117,118]. Due to post-vaccination complications, vaccines from this group were withdrawn from the market in these countries and were never used again. The negative scenario related to reversion to a virulent strain was also observed with vaccines against porcine reproductive and respiratory disease. Previously, PRRSV-modified live vaccines have reverted to virulence under farm conditions [119,120,121]. Ever since the first licensed PRRSV vaccine (Ingelvac PRRSR MLV) started to be widely used on swine farms in China and the United States, field isolates from later PRRSV outbreaks in these countries have exhibited nearly identical nucleotide sequences to the vaccine strains [119,122].

The study by Chen et al. [59] also showed that the HLJ/18-6GD strain, in which six genes were deleted (MGF505-1R, MGF505-2R, MGF505-3R, MGF360-12L, MGF360-13L, and MGF360-14L), may become more virulent during replication in pigs. For example, all five pigs administered HLJ/18-6GD after passage 6 showed significant amounts of viral DNA copies in their blood and organs. HLJ/18-6GD demonstrated the potential to become more virulent during its replication, which is undoubtedly alarming. The mechanisms by which the HLJ/18-6GD strain became virulent have yet to be elucidated. More research is needed on these mechanisms as the reversion to lethal vaccine strains would lead to significant economic losses, and the elimination of this scenario is a key safety component for the large-scale use of attenuated vaccines. Additionally, the possibility of infection of vaccinated animals with an ASFV field strain cannot be excluded. The inclusion of genes encoding virulence in this field strain into the genome of the vaccine strain may enhance the virulence of the vaccine strain and disease in vaccinated pigs [24].

Despite the benefits of deleting virulence genes, deletion of genes responsible for virulence from the genome of a vaccine strain candidate in most cases results in a simultaneous weakening of its immunogenicity, which may be related to mutual interactions in the genome. Thus, by obtaining a vaccine without the risk of causing side effects, a biopreparation of low effectiveness can be obtained [123,124].

### 5.5. DIVA Strategy

The application of a vaccine against ASF in the field is dependent on the availability of an accompanying discriminatory test (DIVA test) allowing for differentiation between vaccinated and infected animals. The lack of a DIVA strategy is now another obstacle to the elimination of ASF through immunization. It is also a necessary condition for the official registration of potential vaccines against ASF. The general DIVA principle is based on a DIVA vaccine producing an antibody response that is different from the antibody response produced by the wild-type pathogen [125]. In a DIVA strategy, the vaccine is usually accompanied by a diagnostic test to distinguish post-vaccination antibodies from those directed against the wild-type pathogen. Another type of DIVA strategy is the so-called ‘genetic’ DIVA, which is based on identifying genetic differences between live vaccines and the field viruses [125] A potential product used in the field must satisfy a variety of requirements, including efficacy and the use of markers that differentiate infected from vaccinated animals [17]. The DIVA technique allows us to distinguish animals vaccinated against ASF from animals infected with the field strain and is necessary in the case of effective monitoring of the confirmation of the disease’s eradication within the context of ASF. To ensure proper monitoring and control of the effect of vaccination on disease progression in vaccinated pigs, an ideal vaccine will need both positive and negative markers to reliably distinguish vaccinated from naturally infected animals [126] The tests for a DIVA strategy should be elaborated in parallel with vaccine development. A test for LAVs could be based on those genes that have been deleted to provide a DIVA test based on a negative marker (the potential negative marker should be assessed for induction of antibodies in unvaccinated, infected animals that would be absent in vaccinated animals) [126]. Positive selection may also be considered, as markers present in the genome of genetically manipulated strains can also be detected by molecular or serological methods [126]. High hopes regarding DIVA are attributed to subunit and vectored vaccines with many immunodominant antigens allowing for differential diagnostics [7,46]. The viral vectors appear to be optimal for a DIVA strategy, since the immunogens encoded in viral vectors can serve as vaccine markers [70]. African swine fever virus is widespread and developing DIVA vaccines and the corresponding diagnostic tests is necessary to eliminate the virus from the wildlife reservoir. Barasona et al. [61] report the possibility of further progress towards the development of an oral vaccine whose efficacy can be tested in wild boars under field conditions. Goatley et al. [73] published a promising approach, in which immunization and challenge are performed with a pool of eight ASFV antigens, in terms of antigen choice and demonstrated that a DIVA-compatible subunit vaccine could confer protection. The findings of this study have influenced the direction of future research. 

## 6. Conclusions

Due to the biological properties of ASFV, efforts to design an appropriate and effective vaccine against this fatal disease have been made for several decades, although they have proved to be ineffective so far. These efforts have been complicated by many aspects: surviving pigs are protected against infection only with a homologous virus; cross-resistance is difficult to achieve; no definitive immune correlates of protection have been detected to date; and the viral antigens involved in immune protection against infection with virulent ASFV remain unknown. At present, the greatest expectations lie in recombinant live attenuated vaccines and research advances in the sector of subunit vaccines, DNA vaccines, and vector vaccines. Recent, promising results provide hope for the implementation of an effective vaccine against ASFV. The problem in most European countries concerns the large contribution of wildlife to the spread of the virus; hence, the design of oral vaccines seems to be the most appropriate solution.

## Figures and Tables

**Table 1:** Cases and outbreaks of ASF in 2021 [16].

Region	Country	Animal Category
Africa	Namibia	Domestic
	Nigeria	Domestic
	Sierra Leone	Domestic
	South Africa	Domestic
	Tanzania	Domestic
	Zambia	Domestic
Asia	China (People’s Rep. of)	Domestic and wild
	Hong Kong	Domestic
	India	Domestic
	Indonesia	Domestic
	Korea (Dem People’s Rep. of)	Domestic
	Korea (Rep. of)	Domestic and wild
	Laos	Domestic
	Malaysia	Domestic and wild
	Myanmar	Domestic
	Philippines	Domestic
	Timor-Leste	Domestic
	Vietnam	Domestic
Europe	Bulgaria	Domestic
	Estonia	Domestic and wild
	Germany	Wild
	Greece	Domestic
	Hungary	Wild
	Latvia	Wild
	Lithuania	Domestic and wild
	Moldova	Domestic and wild
	Poland	Domestic and wild
	Romania	Domestic and wild
	Russia	Domestic and wild
	Serbia	Domestic and wild
	Slovakia	Domestic and wild
	Ukraine	Domestic and wild
Oceania	Papua New Guinea	Domestic

**Table 2:** Promising vaccines against ASFV developed in 2015–2021.

ASFV Strain	Virulence	Genotype (P27)	Deleted Genes	Deletion Mutant	Effects	Reference
Georgia 2010		II	I177L	ASFV-G-ΔI177L	full attenuation in pigs	[57]
Georgia 2010		II	I177L	ASFV-G-ΔI177L	Oronasal administration is as effective as i.m. administration, animals remained clinically healthy after challenge with ASFV-G, and the specific antibody response was on the same level	[58]
HLJ/18	High	II	Gene segments MGF505-1R, MGF505-2R, MGF505-3R, MGF360-12L, MGF360-13L, MGF360-14L	HLj/18-7GD	full attenuation in pigs, assurance of complete immunity against lethal ASFV challenge, unable to be converted to the virulent strain	[59]
OURT 88/3	Low	I	I329L	OURT88/3ΔI329L	inhibits the host’s innate immune response,	[60]
L v17/WB/Rie1217		II			92% protection in wild boars after challenge with the virulent ASF virus isolate Arm07	[61]
Georgia 2007	High	II	9GL (B119L) UK (DP96R)		full attenuation in pigs	[62,63]
